# Ultrasound-guided erector spinae plane block for postoperative short-term outcomes in lumbar spine surgery: A meta-analysis and systematic review

**DOI:** 10.1097/MD.0000000000032981

**Published:** 2023-02-17

**Authors:** Hui Liu, Jing Zhu, Jing Wen, Qiang Fu

**Affiliations:** a Department of Anesthesiology, The Third People’s Hospital of Chengdu, Chengdu City, China.

**Keywords:** erector spinae plane block, lumbar spine surgery, meta-analysis, postoperative analgesia, short-term outcome, ultrasound-guided

## Abstract

Patients undergoing lumbar spine surgery usually suffer from moderate to severe acute pain. Erector spinae plane block (ESPB) has been applied to relieve acute pain in various surgeries and improve postoperative outcomes. This study aimed to further identify the efficacy and safety of erector spinae plane block in patients undergoing lumbar spine surgery. This study also evaluates the outcomes of the erector spinae plane block compared with other regional blocks.

**Methods:**

We searched PubMed, Web of Science, Cochrane library, Embase, and CINAHL databases to identify all randomized controlled trials evaluating the effects of ESPB on postoperative pain after lumbar spine surgery. The primary outcome is postoperative total opioid consumption in 24 hours. The secondary outcomes are postoperative pain scores, intraoperative opioid consumption, time to first rescue analgesia, number of patients requiring rescue analgesia, first time to ambulation after surgery, length of hospital stay, patients’ satisfaction score, and postoperative side effects such as postoperative nausea and vomiting, itching.

**Results:**

A total of 19 randomized controlled trials are included in the final analysis. Compared with no/sham block, ultrasound-guided erector spinae plane block can decrease perioperative opioid consumption including intraoperative opioid consumption: standardized mean difference (SMD) = −3.04, 95% confidence interval (CI) (−3.99, −2.09), *P* < .01, and opioid consumption postoperatively: (SMD = −2.80, 95% CI [−3.61, −2.00], *P* < .01); reduce postoperative pain at 2, 6, 12, 24, and 48 hours both at rest and movement; meanwhile shorten time to hospital length of stay: (SMD = −1.01, 95% CI [−1.72, 0.30], *P* = .006), decrease postoperative nausea and vomiting (RR = 0.35, 95% CI [0.27, 0.46], *P* < .00001), and improve patient satisfaction (SMD = −2.03, 95% CI [−0.96, 3.11], *P* = .0002). But ultrasound-guided ESPB doesn’t shorten the time to ambulation after surgery (SMD = −0.56, 95% CI [−1.21, 0.08], *P* = .09). Additionally, ESPB is not superior to other regional blocks (e.g., thoracolumbar interfascial plane/midtransverse process to pleura block).

**Conclusion:**

This meta-analysis demonstrates that ultrasound-guided ESPB can provide effective postoperative analgesia in patients undergoing lumbar spine surgery and improve postoperative outcomes, and it deserves to be recommended as an analgesic adjunct in patients undergoing lumbar spine surgeries.

## 1. Introduction

More and more lumbar spine surgeries have been performed in the past few decades. Postoperative moderate to severe pain is frequently encountered in patients following lumbar spine surgery and the acute pain after surgery was often controlled poorly.^[1–3]^ Postoperative pain is associated with delayed postoperative ambulation, venous thrombosis, and an increased incidence of pulmonary and cardiac complications, which may delay postoperative recovery, and even prolong the length of hospital stays.^[4]^ In addition, inadequate acute pain treatment may lead to postoperative persistent pain.^[5]^ Therefore, it is imperative to effectively manage postsurgical pain following lumbar spine surgery. Patient-controlled intravenous analgesia (PCIA) with opioid analgesics is the most common analgesic method for kinds of surgeries and can provide excellent pain relief. However, high-dose opioids are limited by side effects like nausea, vomiting, respiratory inhibiting, pruritus, addiction, and other opioid-related side effects.^[6]^ Furthermore, the opioid crisis has been a public health challenge.^[7]^

Multimodal analgesia has been the recognized mode for postoperative analgesia and is the direction of future research. Ultrasound-guided fascial plane blocks have as become an essential part of multimodal analgesia because of the simplicity of operation and fewer complication*s*.^[8]^ The erector spinae plane block (ESPB) was first used in chronic thoracic neuropathic pain by Forero in 2016.^[9]^ Over the past several years, it was widely used in different types of surgeries, such as breast surgery,^[10]^ thoracic surgery,^[11]^ and laparoscopic cholecystectomy,^[12]^ and achieved good analgesic effectiveness. Recently, several systematic reviews and meta-analyses reported that ESPB can provide effective analgesia in lumbar surgery. But most of the studies included a small number of randomized controlled trials (RCTs) and all of them did not compare ESPB with other types of blocks. For example, Liu’s meta-analysis revealed that ESPB was effective and safe for postoperative analgesia, but only six RCTs were included and it did not pay attention to some important outcomes, such as pain score at rest and length of hospital stay.^[13]^ Additionally, Oh’s review incorporated some abstracts and letters without sufficient RCTs.^[14]^ This study, therefore, aimed to further clarify the efficacy and safety of ESPB on postoperative analgesia outcomes in patients undergoing lumbar spine surgery. It also evaluated the analgesic effect of erector spinae plane block compared with other regional block techniques.

## 2. Methods and Materials

The present study is a systematic review and meta-analysis of previously published RCTs, so ethical approval was not required. This study was conducted and reported in line with preferred reporting items for systematic review and meta-analysis guidelines.^[15]^ PROSPERO registration number: CRD42021284430.

### 2.1. Search strategy and study selection

The following databases were searched (from inception to March 2022): PubMed, Web of Science, Cochrane library, Embase, and CINAHL Keywords including “lumbar spine surgery,” “decompression,” “spondylolisthesis,” “lumbar spinal stenosis,” “ESP block,” “ESPB,” “erector spinae plane block” and “erector spinae block” were used with no limits on language. To prevent missing any relative research, references to relevant articles or reviews and meta-analyses were also screened and checked. We included published full-text randomized controlled trials involving adults undergoing lumbar spine operations following ESPB with no block, sham block, or other regional blocks. Case reports, ongoing trials, articles without completed results, abstracts, letters, comments, or editorials were not considered for inclusion. Non-ultrasound-guided regional blocks were also excluded.

### 2.2. Data extraction

Firstly, two authors browsed the title and abstract to exclude irrelevant literature. Then we further reviewed the full text of the literature that initially met the inclusion criteria. Any inconsistencies were discussed with a third author. Two reviewers extracted separately the data using a data collection table. The following items were recorded: the first author, publication year, age, sample size, surgery description, the use of local anesthetics, time, methods of postoperative analgesia, and related outcomes. The primary outcome is postoperative opioid consumption in the first 24 hours. Secondary outcomes include pain score at rest and movement at 2, 6, 12, 24, and 48 hours after surgery, time to first rescue analgesia, the number of patients who requires rescue analgesia, time to ambulation after surgery, length of hospital stay, satisfaction score, opioid-related adverse effects such as incidence of postoperative nausea and vomiting (PONV), itching, etc, and complications associated with blocks.

### 2.3. Quality assessment

Two authors independently assessed the risk of bias in each reviewed study. Any disagreements were resolved by discussion with a third author. The Cochrane Collaboration risk of bias assessment was used to assess the quality of each study.^[16]^ The risk of bias was classified as low, unclear, or high risk. We evaluated the quality of evidence of primary outcome using the Grading of Recommendations, Assessment, Development, and Evaluation guidelines.^[17]^

### 2.4. Statistical analysis

Statistical analysis was conducted using Review Manager (RevMan 5.3). For continuous data, standardized mean differences (SMD) with a 95% confidence interval (CI) were calculated. The dichotomous data, Risk ratios (RR) with 95% CI were calculated. If the data were shown as median, minimum to maximum or inter-quartile ranges, we converted them to mean and standard deviation.^[18,19]^ When data were presented as a graph, we extracted the information with digitizing software (GetData Graph Digitizer 2.26). We tried to contact the corresponding author to obtain the raw data if data could not be found in a study. Random-effects modeling was used to pool data. When a *P* value < .05, it was considered statistically significant. We used *I*^2^ statistics to check the heterogeneity of each study and *I*^2^ > 50% meant significant heterogeneity. We performed sensitivity analysis and subgroup analysis to evaluate the stability of the results and explored the possible sources of heterogeneity. The Egger test by Stata 12.0 was used to assess potential publication bias.

## 3. Results

### 3.1. Literature search, study characteristics, and quality assessment

**Figure 1.**
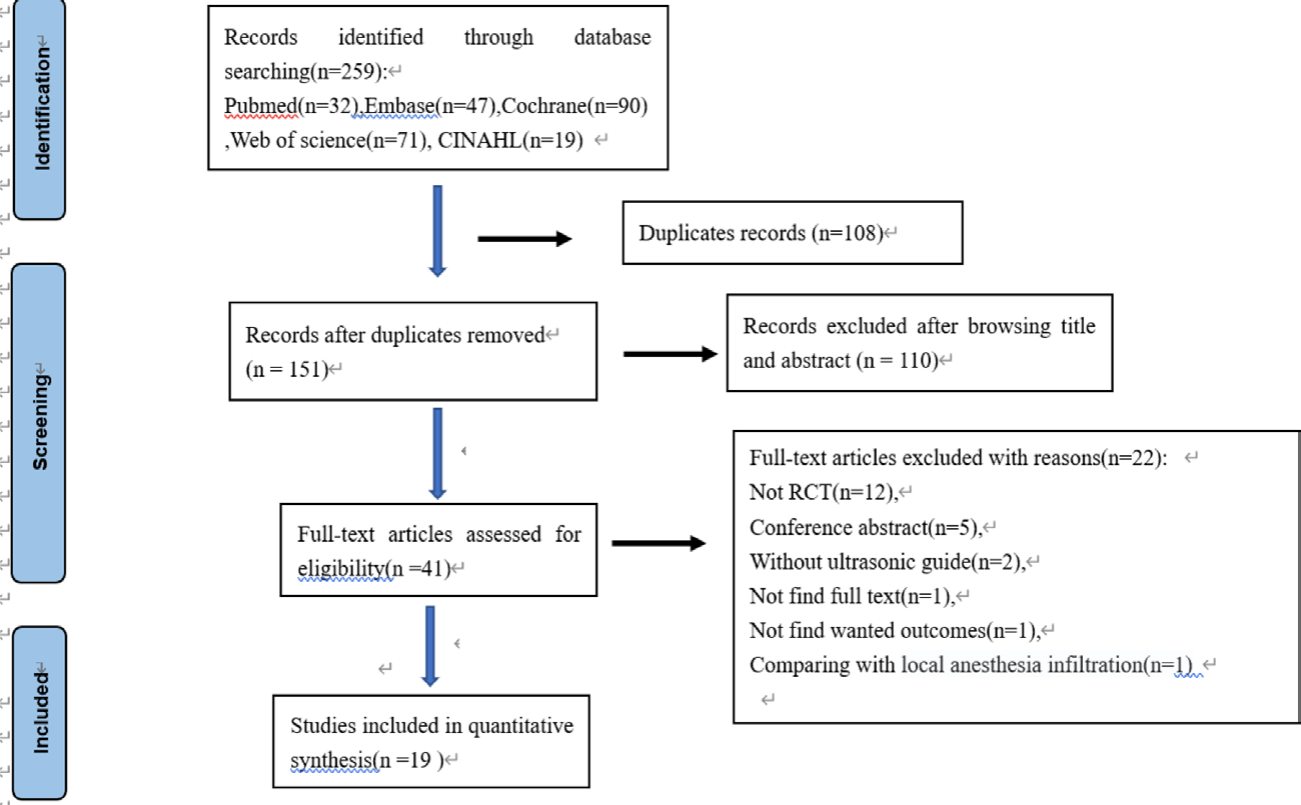
Flow diagram of study searching and selection process. RCT = randomized controlled trial.

**Figure 2.**
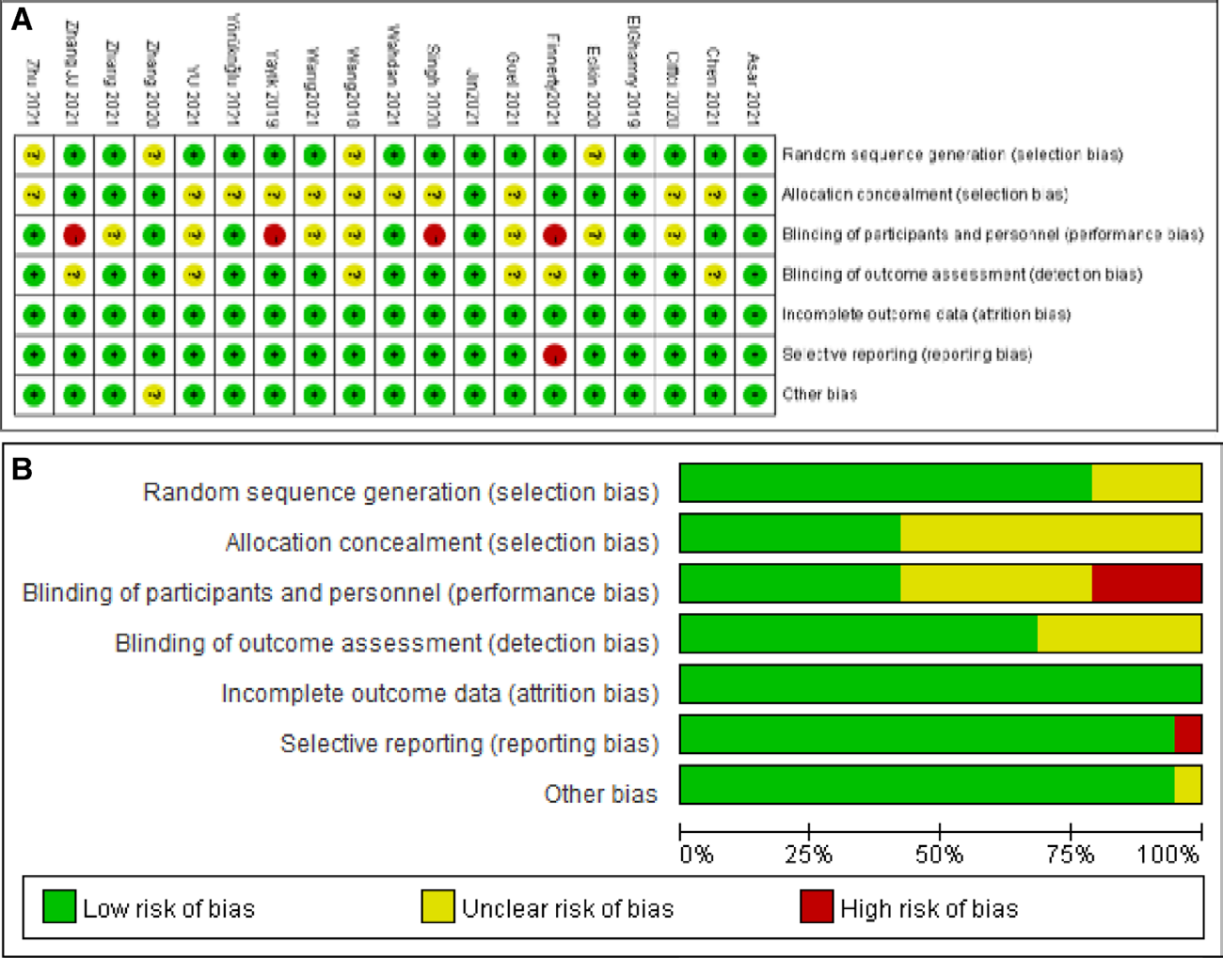
(A) Risk of bias summary: review authors’ judgements about each risk of bias item for each included study; (B) Risk of bias graph: review authors’ judgements about each risk of bias item presented as percentages across all included studies.

The initial search identified 259 studies. After removing 108 duplicated studies, we excluded irrelevant studies by browsing titles and abstracts. Subsequently, 41 full-text articles were further acquired and evaluated. Then 20 randomized controlled trials (RCTs) were assessed for eligibility and one article was excluded because of a comparison with local anesthetic infiltration.^[20]^ Finally, 19 articles were included in this systematic review and meta-analysis.^[21–39]^ The detailed screening process and selection results are shown in Figure 1. The characteristics of the included 19 RCTs enrolling in 1561 patients are shown in Table 1. Nineteen trials compared ESPB with no block or sham block, among two trials compared ESPB both to no block and thoracolumbar interfascial plane (TLIP) block,^[26,29]^ and one trial^[22]^ compared ESPB both to no block and midtransverse process to pleura (MTP) block. We could not get access directly to the primary data in the study of Eskin et al,^[22]^ so we got in touch with the authors to get the relevant data. ESPB is performed by ultrasound guidance with a single injection of local anesthetics in all studies. Bupivacaine or levobupivacaine were administered in ten studies,^[21–24,29–33,35]^ ropivacaine was administered in eight studies,^[25–28,34,37–39]^ and the mixture of bupivacaine and lidocaine solution was administered in one study.^[36]^ The risk of bias for each included study is summarized in Figure 2. Fifteen studies reported specific randomization methods and allocation concealment is adequate in only eight studies. The risk of performance bias was classified as high in four RCTs and most studies were judged to be at low risk for detection bias, selective reporting, and other biases. Quality of evidence for the opioid consumption in the first 24 hours after surgery was downgraded to low due to the high heterogeneity and risk of bias. Egger test by Stata 12.0 was performed to evaluate publication bias and revealed there was no obvious publication bias (*P* = .186 > .05).

**Table 1:** Characteristics of the included studies.

Study and year	Age (yr)	Group and number	Procedures	Treatments	Time	Perioperative analgesia	Postoperative analgesia	Rescue analgesia
1. Ciftci 2020^[29]^	18–65	ESPB/mTLIP/control (30/30/30)	Single-level lumbar discectomy and hemilaminectomy surgery	Ultrasound-guided ESPB group: 20 mL of 0.25% bupivacaine (each side) Ultrasound-guided mTLIP group: 20 mL of 0.25% bupivacaine (each side) Control group: no block.	After the induction of anesthesia	Remifentanl	Paracetamol + PCIA (fentanyl)	Meperidine iv (VAS ≥ 4)
2. Eskin 2020^[22]^	18–80	ESPB/MTP/control (40/40/40)	Elective lumbar decompression surgery	Ultrasound-guided ESPB group: 20 mL of 0.25% bupivacaine (each side) MTP block: 20 mL of 0.25% bupivacaine (each side) Control group: no block	After the induction of anesthesia	Remifentanil	Paracetamol + dexketoprofen + PCIA (Tramadol)	Pethidine iv (VAS > 3)
3. Zhang 2020^[25]^	18–80	ESPB/control (30/30)	Open posterior lumbar decompression surgery	Ultrasound-guided ESPB group: 25 mL of 0.3% ropivacaine (each side) Control group: no block	Before the induction of anesthesia	Sufentanil	PCIA (Morphine)	
4. Yayik 2019^[24]^	18–65	ESPB/control (30/30)	Open lumbar decompression surgery	Ultrasound-guided ESPB group: 20 mL of 0.25% bupivacaine (each side) Control group: no block	Before the induction of anesthesia	Remifentanil	PCIA (Tramadol)	Pethidine (VAS ≥ 4)
5. Singh 2020^[23]^	18–65	ESPB/control (20/20)	Prolapsed lumbar intervertebral disk, lumbar stenosis or laminectomy	Ultrasound-guided ESPB group: 20 mL of 0.5% bupivacaine (each side) Control group: no block	Before the induction of anesthesia	Fentanyl	Intravenous diclofenac	Morphine (NRS ≥ 4).
6. Ghamry 2019^[21]^	18–60	ESPB/control (30/30)	Elective PLIF	Ultrasound-guided ESPB group: 20 mL of 0.25% bupivacaine (each side) Control group: 20 mL normal saline (each side)	Before the induction of anesthesia	Fentanyl	Intravenous paracetamol + ketorolac	Morphine iv (VAS > 30)
7. Zhang 2021^[34]^	20–75	ESPB/control (30/30)	Open posterior lumbar spinal fusion surgery	Ultrasound-guided ESPB group: 20 mL 0.4% ropivacaine (each side) Control group: Sham blocks (subcutaneous infiltration: 1% lidocaine 1 mL on each side)	Before the induction of anesthesia	Remifentanil	PCIA (sufentanil) + flurbiprofen	Intravenous sufentanil bolus via PCA (NRS ≥ 4)
8. Wahdan 2021^[31]^	18–65	ESPB/control (70/70)	Elective lumbar spine surgeries.	Ultrasound-guided ESPB group: 20 mL of 0.25% levobupivacaine (each side) Control group: 20 mL normal saline (each side)	After the induction of anesthesia	Morphine sulfate	Ketorolac + PCIA (morphine)	Morphine iv (VAS ≥ 4)
9. Finnerty 2021^[30]^	>18	ESPB/control (30/30)	Open thoracolumbar vertebral decompression for degenerative stenosis or trauma	Ultrasound-guided ESPB group: 20 mL of 0.25% levobupivacaine (each side) Control group: no block	After the induction of anesthesia	Oxycodone	Paracetamol + ibuprofen + oxycodone	Intravenous aliquots of oxycodone (VRS > 2)
10. YU 2021^[32]^	26–67	ESPB/control (40/40)	Posterior internal fixation for single-level lumbar fracture	Ultrasound-guided ESPB group: 30 mL of 0.25% bupivacaine (each side) Control group: normal saline30 mL (each side)	After the induction of anesthesia	Remifentanil	PCIA (sufentanil + flurbiprofen)	Pethidine hydrochloride im (NRS > 4)
11. Yörükoğlu 2021^[33]^	18–65	ESPB/control (28/26)	Elective single-level lumbar microdiscectomy	Ultrasound-guided ESPB group: at the surgical level, 20 mL of 0.25% bupivacaine (each side) Control group: sham block (normal saline 20 mL each side)	Before the induction of anesthesia	No describe	PCIA (morphine)	Tenoxicam 20 mg iv (NRS > 3)
12. Zhu 2021^[27]^	45–70	ESPB/control (20/20)	Posterior lumbar fusion surgery	Ultrasound-guided ESPB group: L2 level, 20 mL of 0.375% ropivacaine (each side) Control group: sham block (normal saline 20 mL each side)	Before the induction of anesthesia	Remifentanil	PCIA (oxycodone)	5 μg sufentanil iv (VAS ≥ 4)
13. Zhang JJ 2021^[28]^	18–80	ESPB/control (30/29)	Lumbar spine surgery	Ultrasound-guided ESPB group: 25 mL of 0.3% ropivacaine (each side) Control: no block.	Before the induction of anesthesia	Sufentanil	PCIA (morphine)	
14. Wang 2021^[26]^	/	ESPB/TLIP/control (102/102/102)	Lumbar spine fusion surgery	Ultrasound-guided ESPB group: 30 mL of 0.375% ropivacaine (each side) Ultrasound-guided TLIP group: 30 mL of 0.375% ropivacaine (each side) Control group: no block	After the induction of anesthesia	Remifentanil	PCIA (sufentanil + flurbiprofen)	Sufentanil 5 µg iv (NRS > 5)
15. Wang 2018^[39]^	18-70	ESPB/control (30/30)	Posterior lumbar surgery	Ultrasound-guided ESPB group: 15 mL of 0.5% ropivacaine (each side) Control group: no block	Before the induction of anesthesia	Remifentanil	PCIA (sufentanil)	Parecoxib sodium 40 mg im (VAS > 4)
16. Asar 2021^[36]^	18–75	ESPB/control (35/35)	Spinal surgery with instrumentation involving single or multi levels in the lumbar or thoracic regions	Ultrasound-guided ESPB group: 20 mL volume consisting of 10 mL of 0.5% bupivacaine, 5 mL of 2% lidocaine, and 5 mL of 0.9% NaCl Control group: no block	At the end of surgery	Remifentanil	PCIA (tramadol) + paracetamol	75 mg diclofenac sodiumim (VAS > 4)
17. Chen 2021^[38]^	30–65	ESPB/control (20/20)	Posterior lumbar interbody fusion	Ultrasound-guided ESPB group: L2 level, 20 mL of 0.375% ropivacaine (each side) Control group: sham block (NS 20 mL each side)	Before the induction of anesthesia	Remifentanil	PCIA (oxycodone)	Sufentanil 5 µg iv (VAS > 4)
18. Goel 2021^[35]^	18–78	ESPB/control (50/50)	Single-level transforaminal lumbar inter-body fusion surgery	Ultrasound-guided ESPB group: 20 mL of 0.25% bupivacaine (each side) Control group: no block	After the induction of anesthesia	Fentanyl	Multimodal analgesia: Paracetamol + ketorolac + pregabalin capsule	Fentanyl (NRS > 5)
19. Jin 2021^[37]^	/	ESPB/control (30/32)	Lumbar laminoplasty	Ultrasound-guided ESPB group: 20 mL of 0.375% ropivacaine (each side) Control group: no block	After the induction of anesthesia	Sufentanil/remifentanil	PCIA (sufentanil, dezocine, dex-medetomidine and palonosetron)	Sodium parecoxib and intramuscular pethidine

ESPB = erector spinae plane block, MTP = midtransverse process to pleura, NRS = numerical rating scale, PCIA = patient-controlled intravenous analgesia, TLIP = thoracolumbar interfascial plane, VAS = visual analogue scale, VRS = Verbal Rating Scale.

### 3.2. Primary outcome

For the primary outcome, total opioid consumption in the first 24 hours after surgery was reported in 18 trials that included 1325 patients.

**Figure 3.**
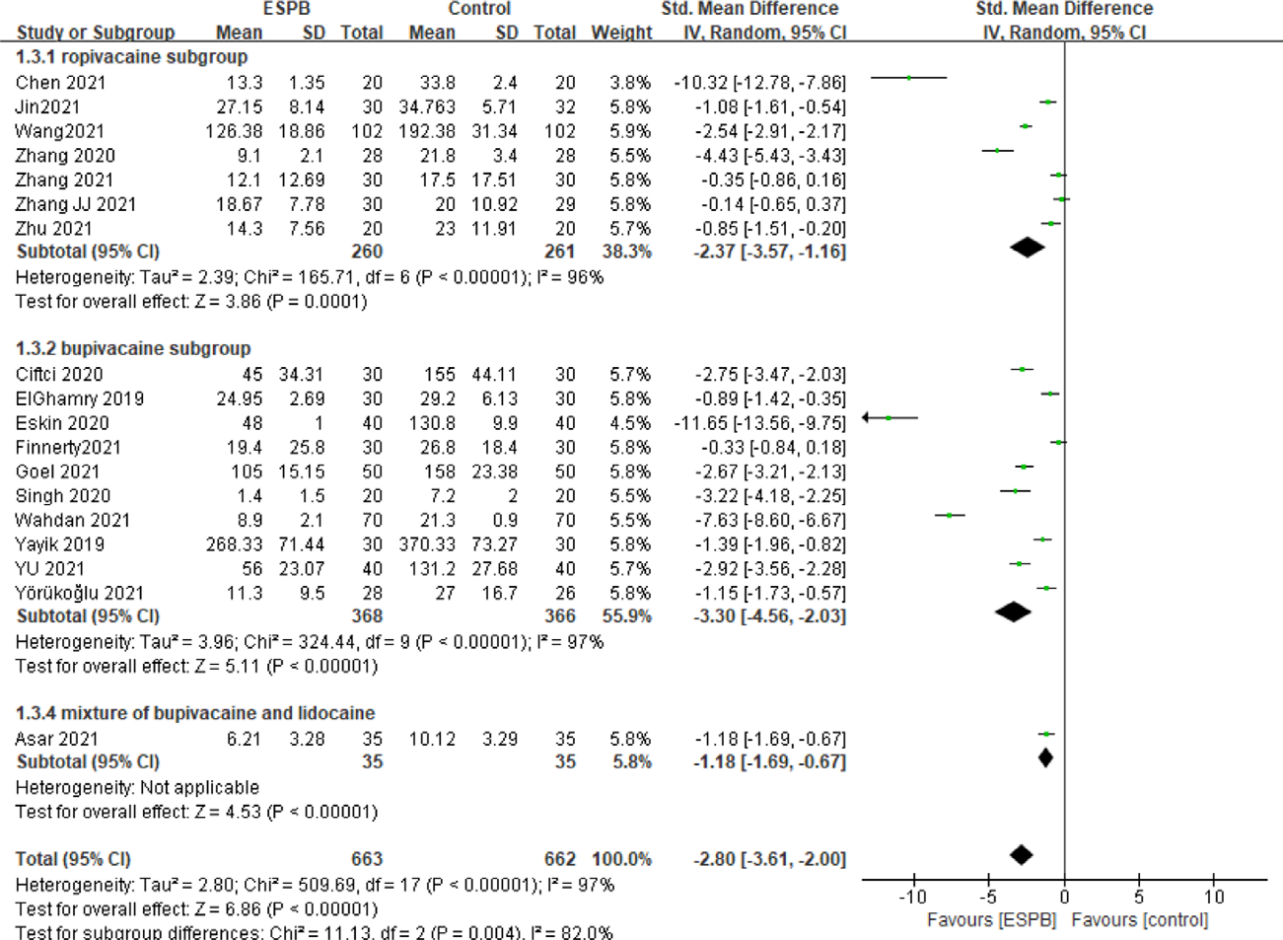
Forest plot for the comparison of postoperative opioid consumption in 24 hours in study groups receiving ESPB compared with no/ sham block. CI = confidence interval, ESPB = erector spinae plane block, SD = standard deviation.

**Figure 4.**
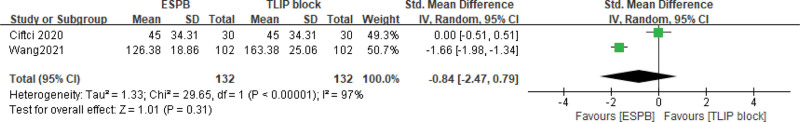
Forest plot of total opioid consumption in first 24 hours after surgery in study groups receiving ESPB compared with TLIP block. CI = confidence interval, ESPB = erector spinae plane block, SD = standard deviation, TLIP = thoracolumbar interfascial plane.

Patients receiving ESPB had significantly decreased postoperative opioid consumption in 24 hours than patients who received no or sham block (SMD = −2.80, 95% CI [−3.61, −2.00], *I*^2^ = 97%, *P* < .00001; Fig. 3). Subgroup analysis was performed according to the type of local anesthetics. In the bupivacaine subgroup, ten studies and 734 participants were analyzed (SMD = −3.30, 95% CI [−4.56, −2.03], *I*^2^ = 97%, *P* < .00001), and in the ropivacaine subgroup, seven studies and 521 patients were included in the analysis (SMD = −2.37, 95% CI = [−3.57, −1.16], *I*^2^ = 96%, *P* < .0001). One study^[36]^ used a mixture of bupivacaine and lidocaine solution and showed opioid consumption was lower in the ESPB group (6.21 ± 3.28) compared to the control group (10.12 ± 3.29) (*P =* .000). According to our sensitivity analysis, there was no change between the primary and sensitivity results after removing one study in turn. So the results were relatively robust and are presented in Table 2. Two studies of ESPB versus TLIP block including 264 patients reported there was no difference in postoperative opioid consumption in 24 hours between the two groups (SMD = −0.84, 95% CI [−2.47, −0.79], *I*^2^ = 97%, *P* = .31; Fig. 4). One study compared ESPB with MTP block and found ESPB can reduce opioid consumption in 24 hours after surgery compared with MTP group (48.0 ± 1.0 vs 84.9 ± 4.0 mg).

**Table 2:** Sensitivity analysis of the primary outcome.

Removed study	SMD	95% CI lower limit	95% CI upper limit	*z* value	*P* value
Asar 2021^[36]^	−2.92	−3.78	−2.06	6.68	<.00001
Chen 2021^[38]^	−2.49	−3.28	−1.71	6.26	<.00001
Ciftci 2020^[29]^	−2.81	−3.65	−1.98	6.59	<.00001
Ghamry 2019^[21]^	−2.94	−3.79	−2.09	6.77	<.00001
ESkin 2020^[22]^	−2.36	−3.09	−1.62	6.29	<.00001
Finnerty 2021^[30]^	−2.96	−3.80	−2.13	6.95	<.00001
Goel 2021^[35]^	−2.82	−3.67	−1.98	6.54	<.00001
Jin 2021^[37]^	−2.93	−3.78	−2.07	6.72	<.00001
Singh 2020^[23]^	−2.78	−3.61	−1.96	6.59	<.00001
Wahdan 2021^[31]^	−2.45	−3.16	−1.74	6.79	<.00001
Wang 2021^[26]^	−2.84	−3.72	−1.97	6.40	<.00001
Yayik 2019^[24]^	−2.91	−3.76	−2.05	6.67	<.00001
YU 2021^[32]^	−2.80	−3.64	−1.97	6.57	<.00001
Yörükoğlu 2021^[33]^	−2.92	−3.77	−2.07	6.73	<.00001
Zhang 2020^[25]^	−2.70	−3.51	−1.89	6.53	<.00001
Zhang 2021^[34]^	−2.96	−3.80	−2.13	6.94	<.00001
Zhang JJ 2021^[28]^	−2.97	−3.80	−2.14	7.03	<.00001
Zhu 2021^[27]^	−2.93	−3.77	−2.09	6.83	<.00001

CI = confidence interval, SMD = standardized mean differences.

### 3.3. Secondary outcome: pain score

**Figure 5.**
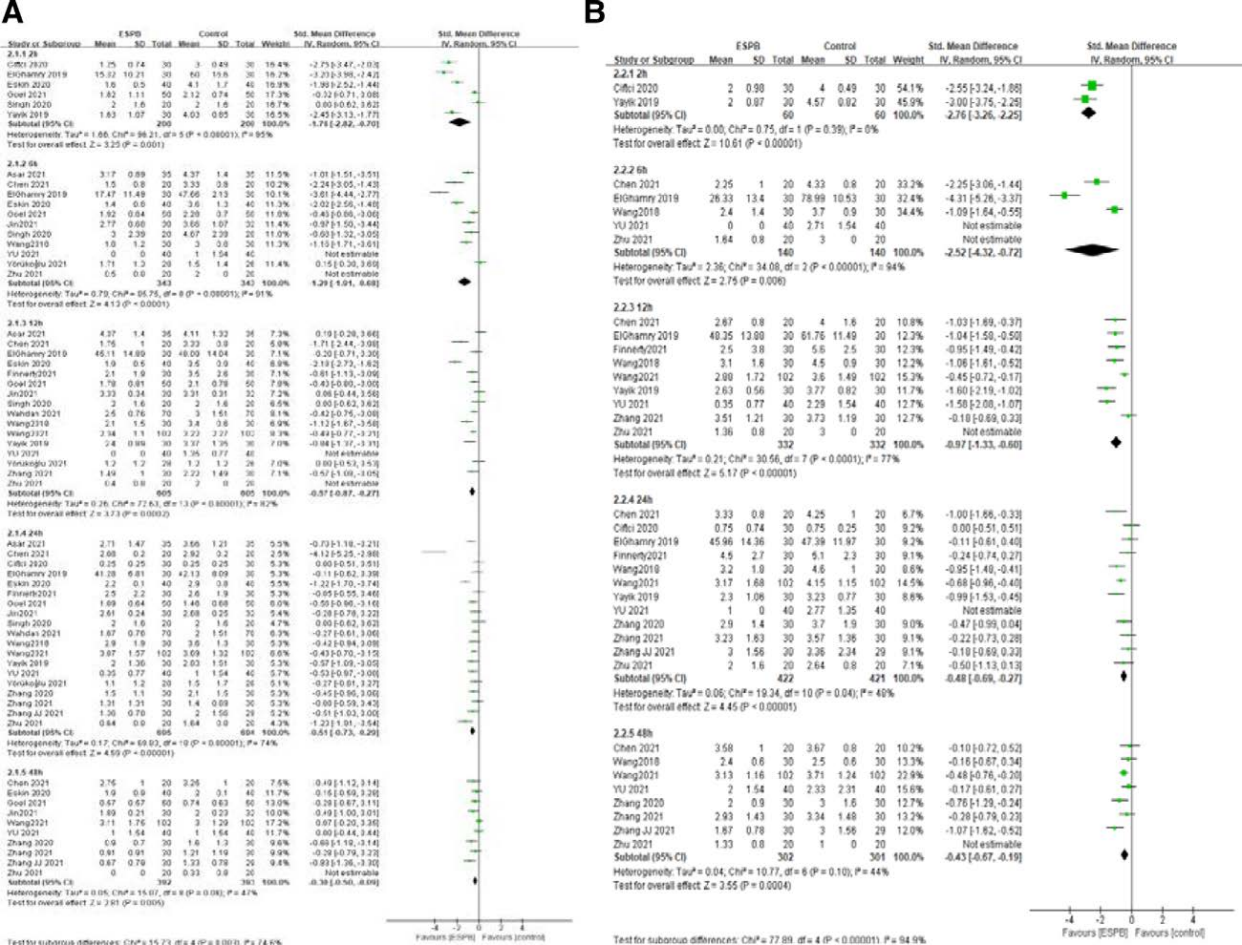
A–B: Forest plot of postoperative pain scores at rest and movement in study groups receiving ESPB compared with no/sham block. (A) Postoperative pain scores at rest in the first 48 hours. (B) Postoperative pain scores at movement in the first 48 hours. CI = confidence interval, ESPB = erector spinae plane block, SD = standard deviation.

**Figure 6.**
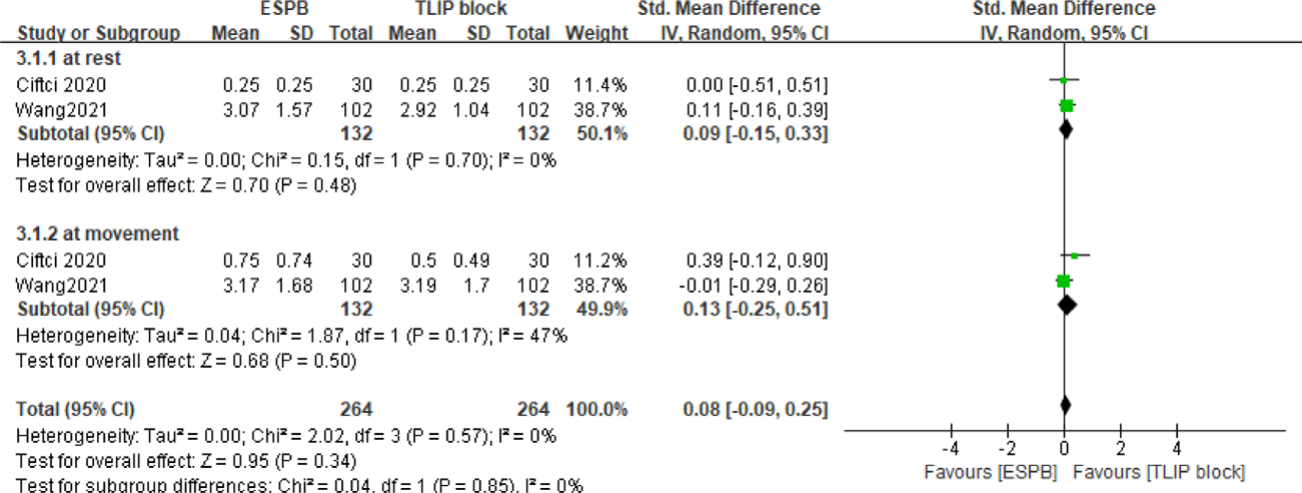
Forest plot of pain score at 24 hours in study groups receiving ESPB compared with TLIP block. CI = confidence interval, ESPB = erector spinae plane block, SD = standard deviation, TLIP = thoracolumbar interfascial plane.

We analyzed pain scores at rest and movement at five-time points in the postoperative period: 2, 6, 12, 24, and 48 hours. Different pain assessment tools were used in eligible studies. Visual analogue scale (VAS, 0–10 scale) was applied to assessed pain score in eight studies,^[22,24,27,29,31,37–39]^ one study used 0–100 VAS,^[21]^ nine studies used 0–10 numerical rating scale,^[23,25,26,28,32–34]^ and one study used a 0–10 Verbal Rating Scale.^[30]^ Postoperative pain scores at rest at 2, 6, 12, 24, and 48 hours was reported in six, eleven, sixteen, nineteen, and ten articles, respectively. ESPB significantly reduced postoperative pain intensity at rest compared with no/sham block: at 2 hours: (SMD = −1.76, 95% CI = [−2.82, −0.70], *I*^2^ = 95%, *P* = .001), 6 hours: (SMD = −1.29, 95% CI [−1.91, −0.68], *I*^2^ = 91%, *P* < .0001), 12 hours: (SMD = −0.57, 95% CI [−0.87, −0.27], *I*^2^ = 82%, *P* = .0002), 24 hours: (SMD = −0.51, 95% CI [−0.73, −0.29], *I*^2^ = 74%, *P* < .00001), 48 hours: (SMD = −0.30, 95% CI [−0.50, −0.09], *I*^2^ = 47%, *P* = .005; Fig. 5A). Postoperative pain scores at movement at 2, 6, 12, 24, and 48 hours were reported in two, five, nine, twelve, and eight articles, respectively. The polled results show that patients with ESPB had lower postoperative pain scores at movement compared with no/sham block: at 2 hours: (SMD = −2.76, 95% CI [−3.26, −2.25], *I*^2^ = 0%, *P* < .00001);6 hours: (SMD = −2.52, 95% CI [−4.32, −0.72], *I*^2^ = 94%, *P* = .006); 12 hours: (SMD = −0.97, 95% CI[−1.33, −0.6], *I*^2^ = 77%, *P* < .00001); 24 hours: (SMD = −0.48, 95% CI [−0.69, −0.27], *I*^2^ = 48%, *P* < .00001); 48 hours: (SMD = −0.43, 95% CI [−0.67, −0.19], *I*^2^ = 44%, *P* = .0004; Fig. 5B). We also analyzed the pain score of ESPB versus TLIP block. The results show pain score at 24 hours postoperatively had no significant difference whether at rest or movement (SMD = 0.08, 95% CI [−0.09, 0.25], *I*^2^ = 0%, *P* = .34, Fig. 6). We did not get the pooled results of other time points due to the lack of enough studies. Ciftci et al^[29]^ reported pain score at 2 hours at rest postoperatively was lower in ESPB group, but it was not different at movement. No study reported pain score at 6 hours postoperatively. Wang et al^[26]^ evaluated pain score at 12 and 48 hours postoperatively, and the scores in the rest of Group ESPB was lower than those in Group TLIP block. However, there was no difference in movement between the two groups. Eskin et al^[22]^ reported VAS scores were lower in group ESPB than group MTP block at postoperative 2, 6, 8, and 12 hours, but there was no difference at postoperative 24, 48 hours.

### 3.4. Other outcomes

Other outcomes of the identified trials are reported in Table 3. Intraoperative opioid consumption was reported in twelve studies that included 452 patients in ESPB groups and 453 patients in the control (no/sham block) groups. Patients undergoing ESPB had lower intraoperative opioid requirements than patients with no/sham block (SMD = −3.04, 95% CI [−3.99, −2.09], *I*^2^ = 96%, *P* < .00001). Two articles reported intraoperative opioid consumption for ESPB versus TLIP block. The results showed no statistical differences between the two groups at intraoperative opioid consumption (SMD = −0.03, 95% CI [−0.21, 0.27], *I*^2^ = 0%, *P* = .79).

**Table 3:** Other outcomes data of RCTs included in the meta-analysis.

Outcomes (ESPB vs no/sham block)	Studies included	RR or Std. mean difference [95% CI]	*P* value for statistical significance	*P* value for heterogeneity	I^*2*^ test for heterogeneity
Intraoperative opioid consumption	12	3.04 [−3.99, −2.09]	<.00001	<.00001	96%
Time to first rescue analgesic postoperatively	5	7.77 [5.25, 10.29]	<.00001	<.00001	94%
Number of patients requiring rescue analgesia	8	0.33 [0.25, 0.43]	<.00001	.55	0%
First time to ambulation after surgery	4	−0.56 [−1.21, 0.08]	.09	<.00001	87%
Hospital length of stay	8	−1.01 [−1.72, −0.30]	.006	<.00001	95%
PONV	13	0.35 [0.27, 0.46]	<.00001	.12	33%
Itching	6	0.48 [0.27, 0.84]	.01	.18	34%
Satisfaction score	4	2.03 [0.96, 3.11]	.0002	<.00001	94%
Outcomes (ESPB vs TLIP block)	Studies included	RR or Std.mean differance [95% CI]	*P* value for statistical significance	*P* value for heterogeneity	*I^2^* test for heterogeneity
Intraoperative opioid consumption	2	0.03 [−0.21, 0.27]	.79	.51	0%
Number of patients requiring rescue analgesia	2	0.90 [0.51, 1.61]	.73	.28	14%
PONV	2	1 [0.56, 1.77]	0.99	0.70	0%

CI = confidence interval, ESPB = erector spinae plane block, PONV = postoperative nausea and vomiting, RCT = randomized controlled trial, RR = risk ratios, TLIP = thoracolumbar interfascial plane.

For erector spinae plane block versus no/sham block, five studies including 380 patients reported time to first rescue analgesic. The results indicated ESPB significantly prolonged the time to first rescue analgesic (SMD = 7.77, 95% CI [−5.25, 10.29], *I*^2^ = 94%, *P* < .00001). The number of patients requiring rescue analgesia was reported in eight studies including 578 patients and was significantly lower in the erector spinae plane block groups compared to no/sham block group (49 patients vs 150 patients, RR = 0.33, 95% CI [0.25, 0.43], *I*^2^ = 0%, *P* < .00001). One article^[22]^ reported time to first rescue analgesic and showed that patients receiving ESPB prolonged time to first rescue analgesic compared to MTP block group (14.2 ± 1.6 hour vs 0.8 ± 0.4 hour).

Two studies of ESPB versus TLIP block reported the number of patients requiring rescue analgesia and there was no significant difference between the two groups (21 patients vs 24 patients, *P* = .73). One article^[22]^ recorded ESPB compared with MTP block and reported the number of patients requiring rescue analgesia was significantly lower in the group ESPB(7 patients) than the group MTP block (22 patients).

Five studies including 309 patients recorded first time to ambulation after surgery and there was no significant difference between patients who received ESPB and those who received no/sham block (SMD = −0.56, 95% CI [−1.21, 0.08], *I*^2^ = 87%, *P* = .09).

Eight studies that included 367 patients in ESPB groups and 369 patients in no/sham block groups measured the hospital length of stay and it was significantly shorter in patients with ESPB (SMD = −1.01, 95% CI [−1.72, 0.30], *I*^2^ = 95%, *P* = .006). One article^[26]^ reported that there was a similar hospital stay between ESPB and TLIP block.

Fifteen studies that included 1006 patients compared the incidence of PONV in patients receiving ESPB with no/sham block and found the ESPB significantly reduced the incidence of PONV compared with no/sham block (RR = 0.35, 95% CI [0.27, 0.46], *I*^2^ = 33%, *P* < .00001). Two studies including 264 patients of ESPB versus TLIP block showed there was no difference in the incidence of PONV (RR = 1.00, 95% CI [0.56, 1.77], *I*^2^ = 0%, *P* = .99). One article^[22]^ reported the incidence of PONV was lower in patients with ESPB compared to MTP block (1(2.5%) vs 4 (10%)).Six studies compared the incidence of itching in patients receiving ESPB with no/sham block. ESPB reduced the incidence of itching after surgery compared with no/sham block (RR = 0.48, 95% CI [0.27, 0.84], *I*^2^ = 34%, *P* = .01). One study^[29]^ reported patients receiving TLIP block were lower in the rate of itching compared with those receiving ESPB (7 [23.3%] vs 4 [13.3%]). The study of Eskin et al^[22]^ reported that fewer patients in the group ESPB suffered from itching compared to group MTP block (1 [2.5%] vs 3 [7.5%]). Four studies including 360 patients compared satisfaction scores of patients receiving ESPB with no/sham block. Patients receiving ESPB had higher postoperative satisfaction scores (SMD = −2.03, 95% CI [−0.96, 3.11], *I*^2^ = 94%, *P* = .0002). Eskin et al^[22]^ reported patient satisfaction scores were higher in the group ESPB than the group MTP block (8.2 ± 1.4 vs 7.1 ± 0.9). No complications associated with nerve blocks such as pneumothorax, infection, and local anesthetic toxicity were reported in all included studies.

## 4. Discussion

Our study demonstrates that ultrasound-guided erector spinae plane block preoperatively is a feasible and effective adjunct for acute pain control after lumbar spine surgery. Compared with no/sham block, erector spinae plane block preoperatively not only reduces intraoperative and postoperative opioid consumption in 24 hours but also decreases postoperative pain scores at all measured time points up to 48 hours. In addition, ESPB can reduce opioid-related side effects, improve patient satisfaction, and shorten hospital stays. However, there was no sufficient evidence to support ultrasound-guided ESPB was better than other regional blocks.

Currently, the mechanisms of ESPB have not yet been clarified fully. Most clinical studies thought ESPB provides analgesia by blocking the ventral and dorsal ramus of the spinal nerves, or the local anesthetic may diffuse into the paravertebral space even epidural space.^[40]^ The reasons for widely using the ESPB: on the one hand, ultrasound-guided ESPB is easily performed and has fewer complications compared with epidural or paravertebral nerve block; on the other hand, the range of blocking is narrower, therefore it has the advantages of lighter inhibition on respiratory and circulatory function.

The important finding of this meta-analysis is that ESPB preoperatively decreased intraoperative and postoperative opioid consumption in patients with lumbar procedures, which may be beneficial to the long-term outcome for people after lumbar surgery. Perioperative opioid consumption has been associated with tolerance, hyperalgesia, poor pain outcomes, and even more complications.^[41]^ The higher dose and the longer use of postoperative opioids lead to lower satisfaction, a higher risk of prolonged opioid use, and disability after lumbar spine surgery.^[42]^ Therefore, reducing the opioid dosage as far as possible could promote cessation of opioids, which may improve patient postoperative outcomes. Several studies have reported that ESPB had efficacy in the treatment of persistent chronic pain,^[43–46]^ referring to stubborn thoracic neuropathic pain, chronic shoulder pain, and post-herpetic neuralgia. But most of these studies are case reports, case series, and retrospective analyses, lacking high-quality evidence. Further, large sample studies about ESPB are needed to evaluate its efficacy in chronic pain. As we know, preoperative ESPB can prevent the afferent noxious stimulus in advance and reduce central sensitization, thereby reducing intraoperative and postoperative opioid consumption. However, postoperative ESPB does not reduce intraoperative opioids. Additionally, using PCIA or not also impact the consumption of opioids after surgery.

Our study found that ESPB can decrease postoperative pain scores within 48 hours, while previous meta-analyses only analyzed pain scores within 24 hours. A study about freehand ESPB reported there was no difference in pain scores at 24 hours between ESPB group and the control group. But several case series found that there was still a low pain score in 72 hours after surgery. At present, the effect of ESPB on postoperative pain score varies greatly among different studies, possibly because of the type of local anesthetic, dose, adjuvant, and indwelling catheter or not. Ropivacaine and bupivacaine are the main local anesthetics drugs, the concentration of ropivacaine is 0.2% or 0.375%, and bupivacaine is 0.25% or 0.5%. Subgroup analysis showed both drugs were effective analgesics. Furthermore, the duration of postoperative analgesia may be affected by the time of performing the erector spinae plane block(before/after surgery). Most of the included studies performed ESPB before surgery except Asar et al^[36]^ at the end of surgery. Performing erector spinae plane block before surgical stimulation can prevent peripheral and central sensitivity caused by pain and inflammatory stimulation by blocking the dorsal branch of spinal nerves, and play a role in advanced analgesia.

Although our meta-analysis doesn’t report block-related complications, some studies have found that erector spinae plane block is not safe absolutely. Yawata et al^[47]^ reported a case of local anesthetic poisoning after ESPB with a total of 30 mL of 0.5% levobupivacaine. Even though the local drug did not exceed the limit in this case, local drug poisonings occurred. Another study reported transient postoperative paraplegia occurred in a patient with ESPB and it may be due to the local anesthetic diffusing forward into the epidural space.^[48]^ Recently, a retrospective study of 342 consecutive cases analyzed complications associated with ultrasound-guided erector spinae plane block and didn’t find other complications related to ESPB except for one unilateral pneumothorax.^[49]^ Thus, ultrasound-guided ESPB is a relatively safe analgesic method for patients undergoing lumbar spine surgery. However, we should always be alert to related complications such as local anesthetic intoxication, muscle weakness, infection, and hematoma when performing erector spinae plane block or other regional nerve blocks. Moreover, further studies are needed to investigate the changes in plasma concentration of local anesthetics after erector spinae plane block to improve safety. Our meta-analysis also didn’t find some evidence that ESPB reduced postoperative ambulation time. The probable reason is most doctors still recommend complete bed rest to reduce the burden of the lumbar spine for patients undergoing lumbar surgery. The previous meta-analysis about ESPB did not report this important outcome. Our meta-analysis shows that erector spinae plane block shortens the length of the patient’s hospital stay with high heterogeneity. Shortening hospital length of stay is a valuable indicator to evaluate the effectiveness of ESPB and fast recovery, and it also can improve patient satisfaction and reduce financial burden. Less or no opioids, lower postoperative pain scores, and fewer complications are all helpful to shorten the postoperative hospital stay and promote the rapid recovery of patients.

Erector spinae plane block reduces postoperative opioid-related side effects such as nausea and vomiting, and itching, and PONV is a commonly occurring problem after surgical procedures. Although some patients undergoing spinal surgery received appropriate antiemetic therapy, PONV still occurred in about 60% of patients.^[50]^ It not only causes patients to feel unpleasant but also leads to various complications, such as wound breakdown, aspiration pneumonia, bleeding, and hematomas, thus leading to prolonged residence time in the postanesthesia care unit and delay of discharge, even increasing health care costs.^[51]^ The opioid is one of the risk factors for PONV in adults and increases the risk for PONV with a dose-dependent. A meta-analysis by Frauenknecht et al^[52]^ showed that intraoperative opioids correlated with an increased risk of PONV compared with opioid- free anesthesia. Performing of ESPB decreases the rate of PONV, possibly because of the decrease in opioid consumption in our study and our conclusion is consistent with other meta-analyses about ESPB.^[13,53]^

Our meta -analysis also compared erector spinae plane block with other regional blocks, such as TLIP block and MTP block. TLIP block is a new fascial plane block that can block the dorsal branch of the thoracolumbar spinal nerve.^[54]^ The analgesic effect of MTP block is achieved due to the anesthetic drug is deposited at the mid-point between the transverse process and pleura and diffused the paravertebral space to block the spinal nerve root.^[55]^ TLIP block and MTP block both have been shown to reduce postoperative opioid consumption and improve pain in patients with lumbar spine surgeries.^[22,56]^ There are few studies comparing ESPB with other nerve blocks, so the benefits of ESPB compared to other regional techniques remain unclear.

Our pooled results have high heterogeneity. There is still significant heterogeneity after sensitivity analysis is performed for the primary outcome. Several probable reasons can explain the significant heterogeneity: The types, volume, and concentrations of anesthetic drugs are different among including studies (e.g., bupivacaine 0.25%, 0.5%, 20/30 volume; ropivacaine 0.3%, 0.4%, 0.375%, 0.5%, 15/20/25/30 volume). ESPB is performed after induction of anesthesia in ten studies, so dermatomal sensory testing of the block could not be done to exclude possible block failure. Besides, sensory testing of the block was performed before induction of anesthesia only in six studies, the effectiveness of ESPB was uncertain in the rest studies. The detailed surgical methods are different. For example, lumbar spinal surgeries localized one or two levels in most research, but more segments were involved in a few studies. The greater surgical trauma and longer operation time may cause more severe postoperative pain, which leads to a difference in pain scores. Outcomes of some included studies are presented with the median, the first and third quartiles, the range, and even graphs. Conversion of these data to means and standard deviation may introduce errors that could lead to heterogeneity between studies.

This meta-analysis also has some shortcomings. Firstly, some of the included studies lack the double-blind design and detailed allocation concealment, which weaken the quality of the study. Secondly, certain results are associated with significant heterogeneity and sensitivity analysis did not identify the source of heterogeneity. Thirdly, few studies compare ESPB with other types of blocks, so we cannot conclude whether the ESPB was superior to other types of blocks (e.g., TLIP block, MTP block). Fourthly, the number of patients was relatively small in most of the included studies, and more RCTs with larger sample sizes are required to explore this issue further. Finally, some important outcomes such as other complications (hemorrhage, arrhythmia, postoperative respiratory), and postoperative chronic pain are not investigated based on currently limited data and more RCTs should be performed to explore these outcomes.

## 5. Conclusion

Ultrasound-guided erector spinae plane block preoperatively significantly improves the quality of analgesia, decreases opioid consumption, reduces the incidence of PONV, and improves patient satisfaction following lumbar surgery. In summary, the erector spinae plane block could be a valuable adjunct for relieving pain after lumbar surgery. Further studies need to compare the analgesic effect of ESPB with other regional nerve blocks, and pay more attention to the effect of erector spinal block on postoperative chronic pain.

## Author contributions

**Conceptualization:** Hui Liu.

**Data curation:** Jing Zhu, Jing Wen.

**Formal analysis:** Jing Wen.

**Software:** Hui Liu, Jing Zhu.

**Writing – original draft:** Hui Liu.

**Writing – review & editing:** Qiang Fu.
